# Curcumin, Hormesis and the Nervous System

**DOI:** 10.3390/nu11102417

**Published:** 2019-10-10

**Authors:** Maria Concetta Scuto, Cesare Mancuso, Barbara Tomasello, Maria Laura Ontario, Andrea Cavallaro, Francesco Frasca, Luigi Maiolino, Angela Trovato Salinaro, Edward J. Calabrese, Vittorio Calabrese

**Affiliations:** 1 Department of Biomedical and Biotechnological Sciences, University of Catania, Torre Biologica, Via Santa Sofia, 97–95125 Catania, Italy; mary-amir@hotmail.it (M.C.S.); btomase@unict.it (B.T.); marialaura.ontario@ontariosrl.it (M.L.O.); andreacavallaro@tiscali.it (A.C.); calabres@unict.it (V.C.); 2 Fondazione Policlinico Universitario A. Gemelli IRCCS, 00168 Roma, Italy; cesare.mancuso@unicatt.it; 3 Institute of Pharmacology, Catholic University of Sacred Heart, 00168 Roma, Italy; 4 Department of Clinical and experimental Medicine, Division of Endocrinology, University of Catania, 95125 Catania, Italy; f.frasca@unict.it; 5 Department of Medical and Surgery Sciences, University of Catania, 95125 Catania, Italy; maiolino@policlinico.unict.it; 6 Department of Environmental Health Sciences, School of Public Health and Health Science, University of Massachusetts, Amherst, MA 01003, USA; edwardc@schoolph.umass.edu

**Keywords:** hormesis, vitagenes, antioxidants, heme oxygenase

## Abstract

Curcumin is a polyphenol compound extracted from the rhizome of *Curcuma longa Linn* (family *Zingiberaceae*) commonly used as a spice to color and flavor food. Several preclinical studies have suggested beneficial roles for curcumin as an adjuvant therapy in free radical-based diseases, mainly neurodegenerative disorders. Indeed, curcumin belongs to the family of hormetins and the enhancement of the cell stress response, mainly the heme oxygenase-1 system, is actually considered the common denominator for this dual response. However, evidence-based medicine has clearly demonstrated the lack of any therapeutic effect of curcumin to contrast the onset or progression of neurodegeneration and related diseases. Finally, the curcumin safety profile imposes a careful analysis of the risk/benefit balance prior to proposing chronic supplementation with curcumin.

## 1. Introduction

In recent decades, the increasing aging population and the consequent rise in chronic degenerative diseases has led to an augmented investigation of the environmental factors involved in their origin and progression. There is growing evidence indicating that oxidative stress contributes to the etiology and the progression of neurodegenerative diseases, such as Alzheimer’s Disease (AD), Parkinson’s Disease (PD), Amyotrophic Lateral Sclerosis (ALS) and Multiple Sclerosis (MS) [1,2]. From a pathogenetic viewpoint, neurodegenerative disorders are characterized by the progressive loss of neurons in different areas of the central nervous system, leading to cognitive, behavioral, sensory, and motor dysfunctions [3,4,5,6,7,8,9,10,11]. Common pathological features of neurodegenerative diseases are also oxidative stress, accumulation of certain aggregated proteins, depletion of endogenous antioxidant enzyme activity, mitochondrial dysfunction, and neuroinflammation [12]. The link between aging and neuroinflammation has been referred to as “neuro-inflammaging”, which denotes the modulation of a multiple array of genes/proteins: activated microglia and astrocytes increase both nuclear factor-κB (NF-κB) and cyclooxygenase-2 (COX2) and inducible nitric oxide synthase (iNOS) levels; this, in turn, induces the release of pro-inflammatory cytokines, such as interleukin-6 (IL-6), interleukin-1β (IL-1β) and neurotoxic factors, such as reactive oxidative species (ROS) and tumor necrosis factor-α (TNF-α), leading to neuronal death and subsequent cognitive deficits manifested in neurodegenerative diseases [13,14]. Increased NF-κB activation, also trough Toll Like Receptors 4 (TLR4) and the Innate Immune Signal Transduction Adaptor (MYD88), induces release of pro-inflammatory factors, such as TNF-α, IL-1β, IL-6 and iNOS, with a consequent increase in inflammation-mediated signals in a vicious cycle promoting and sustaining neuro-inflammaging. Neuroprotective curcumin, by inducing upregulation of the vitagene system and NF-E2-related factor 2 (Nrf2) and by inhibiting NF-κB activation, is able to break the vicious cycle and hence, limit the progression of the neurodegenerative diseases.

**Figure 1:**
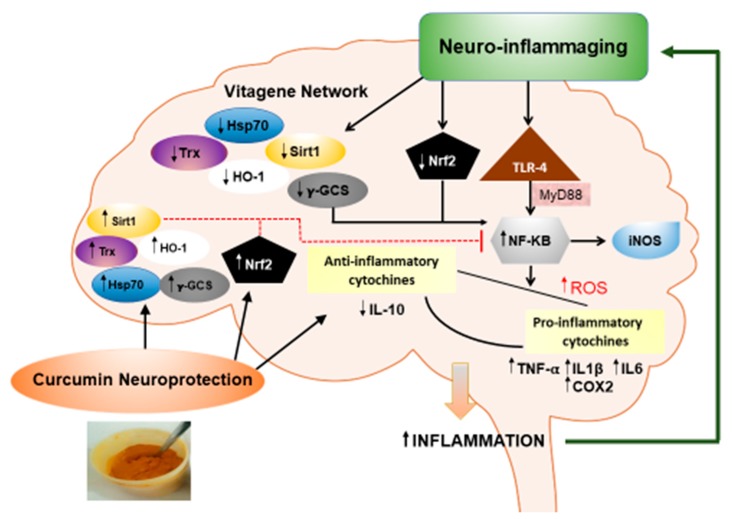
Neuroprotective effects of curcumin on neuro-inflammaging. Neuro-inflammaging is characterized by a down regulation of vitagene system (Hsp 70, γ-GCS, HO-1, Trx and Sirt1) and Nfr2 activity with the consequent upregulation of NF-κB activation. The increased NF-κB activation, also trough Tool Like Receptors 4 (TLR4), induces, in turn, raised proinflammatory factors, such as TNFα, IL1b, IL6, COX2 and iNOS. The disequilibrium between anti-(IL10) and pro-inflammatory molecules leads to increased inflammation, and a vicious circle is established that supports neuro-inflammaging. The neuroprotective curcumin inducing upregulation of vitagene system and Nrf2 could be able to inhibit the NF-κB activation and then break the vicious circle, ending the progression of the neurodegenerative disease.

The heat shock response contributes to maintaining a neuroprotective state against neuroinflammation [1,15]. This adaptive response, which requires the activation of pro-survival pathways as well as the production of molecules endowed with anti-oxidant and anti-apoptotic activities, is reliant upon the control of protective genes known as vitagenes [2,12,16] (Figure 1). The “vitagene” system includes members of the heat shock protein family, such as heme oxygenase-1 (HO-1), heat shock protein (Hsp70), thioredoxin/thioredoxin reductase (Trx/TrxR), sirtuins (Sirt-1) and γ-glutamyl cysteine synthetase (γ-GCS) [17,18,19].

Currently, chronic supplementation with natural products, derived from plants and herbs, in the context of prevention and treatment of neurodegenerative disorders, is considered a new field of investigation [20,21] and preclinical studies have suggested that curcumin could alleviate neuroinflammatory progression [22,23,24]. Although curcumin possesses good therapeutic efficacy in the treatment of different diseases, supplementation with curcumin is difficult due to its poor oral bioavailability, low solubility in aqueous solution, and degradation under physiological conditions [25]. 

In this review, we focus on the evidence leading to the hormetic effects of curcumin, in particular the interaction with the HO-1 system. In addition, the possible advantages of using novel delivery systems of curcumin, which possesses a higher stability and an increased bioavailability than free curcumin, for novel nutraceutical interventions, will be discussed.

## 2. Curcumin

**Figure 2:**
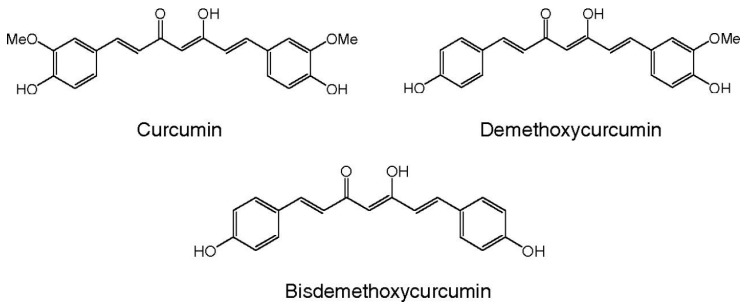
The chemical structure of curcumin and its derivates.

Curcumin, (1,7-bis(4-hydroxy-3-methoxy phenyl)-1,6-hep-tadiene-3,5-dione) is a polyphenol compound extracted from the rhizome of *Curcuma longa Linn* (family *Zingiberaceae*) and it is commonly used in the Asian continent, especially in India, as a spice to color and flavor food. It is also used as cosmetic product, particularly for skin. The curcuminoid complex, found in the rhizome of turmeric (2.5–6%) contains curcumin (CUR) (CUR-diferuloylmethane ~85%), demethoxycurcumin (DEM ~15%), bis-demethoxycurcumin (bis-DEM ~5%), and cyclocurcumin [26]. Curcumin is hydrophobic in nature, so it has poor solubility in water or hydrophilic solutions (Figure 2), while it is highly soluble in organic solvents including methanol, ethanol, acetone and dimethyl sulfoxide [27]. Interestingly, curcumin is a natural fluorophore, with its absorption noted in polar solvents ranging from 408 to 540 nm [28,29].

Curcumin is metabolized to form tetrahydrocurcumin (THC) and hexahydrocurcumin (HHC), together with a small amount of dihydroferulic acid (DHFA) in rats [30]. Dihydrocurcumin (DHC), DEM, *bis*-DEM and the reductive metabolites with glucuronic acid and sulfate were also disclosed, and CUR-glucuronoside, DHC-glucuronoside, THC-glucuronoside and THC were the major metabolites of curcumin in mice [31,32]. Recent studies have suggested that some of the metabolites of curcumin also possess important biological activities. For example, THC could relieve sciatic nerve injury in rats [33] and DHC could prevent the release of glycosaminoglycans induced by antigens in rat basophilic leukemia cell line (RBL-2H3), which may inhibit allergic diseases [34].

Preclinical studies on curcumin have put forth several beneficial effects dealing with its ant-inflammatory, antioxidant, antiproliferative, antiatherosclerosis and antimicrobial properties [35,36,37,38,39,40]. For these reasons, curcumin has been proposed as an adjuvant treatment of several disorders, including diabetes, biliary disorders, spinal cord injury and neurodegenerative diseases [41,42,43,44]. Despite these claimed properties, the poor intestinal absorption, structural instability, limited blood brain barrier (BBB) penetration and rapid degradation of curcumin in the body limits the potential as a therapeutic agent in clinical trials [45]. In addition, an important aspect for the development of curcumin as a novel “nutraceutical’’ formulation deals with obtaining an increased bioavailability and physiological stability and solubility of this compound in animal models and humans [46,47,48]. Studies have attempted to improve bioavailability by altering curcumin’s chemical structure, by conjugating curcumin with lipids, encapsulating curcumin in a nanoparticle, exosomes, constructing complexes with manganese or co-treating curcumin with piperine (Pip) to prevent and treat various neurodegenerative diseases [49,50,51,52,53].

## 3. Curcumin and Brain Targeting

Despite its beneficial effects in vitro, the main obstacle to the medical use of curcumin is its scarce oral bioavailability with a very low plasmatic concentration equal to 1% [54]. In fact, it belongs to the Biopharmaceutics Classification System (BCS) class IV [55].

To date, pharmacokinetic studies have shown that many factors influence the bioavailability of curcumin, including poor water solubility (about 11 ng/mL) [56], chemical instability, limited absorption, fast metabolism and rapid systemic elimination [57]. After oral administration, most of the curcumin is eliminated by fecal excretion while a small quantity is absorbed in the small intestine and is then rapidly converted to water-soluble metabolites in the liver and eliminated by urine [58]. In addition to the aforementioned “bad” pharmacokinetic properties of curcumin, its application in brain diseases raises another critical issue, namely the poor penetration of curcumin across the BBB [59]. A promising therapeutic strategy is represented by novel drug delivery systems, which enable the problems related to curcumin bioavailability to be overcome. New formulations have been developed to achieve this purpose, such as polymeric and lipid nanoparticles, liposomes and cyclodextrins [58].

Recently, preclinical studies have investigated the effectiveness of curcumin nanocarriers in AD models. The administration of solid lipid nanoparticles loaded with curcumin (SLN-Cur, 50 mg/kg) induced a recovery in acetylcholinesterase activity and membrane lipids reduced learning impairment and cognition loss in an aluminum chloride (AlCl3) *mouse model* of AD [60]. Furthermore, some authors have explored the role of poly (lactic-*co*-glicolic acid loaded with curcumin) (PLGA-Cur) in neurogenesis, demonstrating that these nanocarriers were able to stimulate in vitro differentiation and proliferation of neural stem cells as well as improve hippocampal neurogenesis, learning, and memory function in the AD rat model [61]. In another study, glyceryl monooleate nanoparticles loaded with Pip and curcumin (GMO-NP-Pip/Cur) inhibited α-synuclein aggregation and apoptosis. When GMO-NP-Pip/Cur were co-administered with rotenone, motor dysfunction was improved in a PD mouse model [62]. Finally, SLNP-Cur showed neuroprotective effectiveness in a Huntington’s disease rat model by restoring cell redox homeostasis and reducing motor impairment [51]. A current fascinating (interesting) strategy is to trigger curcumin release in the central nervous system (CNS) by the coupling of ligands on the nanoparticle surface, which allows the brain to be targeted rather than crossing the BBB. In this regard, Huang et al. [63] tested PLGA-Cur nanoparticles conjugated with S1 peptide (an inhibitor of β-amyloid generation) and calreticulin (CRT) (ligand of transferrin receptor) in both a cellular model of the (BMEC) (brain microvascular endotelial cells) and a transgenic AD mouse model (APP/PS1dE9). They demonstrated that S1-CRT-NP+Cur can permeate across the BBB and spread in the mouse brain and at the same time, the administration lessened the cognitive deficits, increased the number of synapses, reduced astrogliosis and microgliosis, and decreased inflammation and oxidative stress. Likewise, a neuroprotective effect was exhibited by lactoferin nanoparticles loaded with curcumin (Lf-NP-Cur) in neuroblastoma cell line (SK-N-SH) exposed to rotenone as a PD model [64].

Recently, the delivery to brain of small molecules, proteins and nucleic acids was carried out by exosomes encapsulation [65]. Wang et al. (2019) first demonstrated that exosomes increased the solubility and bioavailability of curcumin. They then showed that the exosomes deriving from curcumin-treated cells (Exo-cur), whose surface is grafted with the endothelial intercellular adhesion molecule 1 (ICAM-1) and the lymphocyte function-associated antigen 1 (LFA-1), enhanced curcumin passage across the BBB through receptor-mediated transcytosis, thus allowing us to prevent neurons dying by inhibiting Tau phosphorylation via the AKT/GSK-3β pathway [53].

Another innovative approach is the intranasal route as it represents a valid treatment route to deliver drugs directly to the brain, bypassing the BBB, which limits the penetration of most of the foreign molecules [65]. In addition, this strategy ensures higher drugs bioavailability and therapeutic efficacy, and less peripheral side effects than oral administration [66,67].

To date, a few studies have tackled the delivery of curcumin to the brain via the direct nose-to-brain route for the treatment of neurodegenerative diseases [67]. Various intranasal delivery systems containing curcumin have been investigated, such as nanoemulsion, thermosensitive poloxamer hydrogel, hyaluronic acid-based lipid nanoemulsion, and microemulsion-based ion sensitive in situ gelling system [68,69,70]. Overall, these preclinical findings suggest that the intranasal route could be an alternative way to deliver drugs to the brain. However, further studies are needed to establish clinical applications of this treatment strategy. Vittorio Calabrese holds a patent (WO2004/075883A1) use of curcumin derivatives or CAPE in the manufacture of a medicament for the treatment of neurodegenerative disorders, and Thomas M. Di Mauro holds two patents (US2008/0076821A1; US 20090326275A1) for intranasal curcumin formulations based on clinical testing on AD patients [67].

Although many efforts are being made to increase curcumin’s bioavailability in order to lead to its clinical application, the efficacy, safety and suitability of these drug delivery systems is still to be clarified.

## 4. Curcumin and Neuroinflammation

Of particular interest to public health and medicine is the potential of curcumin to affect the nervous system. Curcumin (100 mg/kg twice a day for 50 days intragastrically) contrasted extrapyramidal symptoms and increased HO-1 expression through Akt/Nrf2 phosphorylation in the substantia nigra pars compacta of rats treated with rotenone, a pharmacological tool able to destroy dopaminergic neurons and therefore, used to induce experimental Parkinson’s disease (PD) [71]. Curcumin displayed a neuroprotective effect by reducing protein misfolding and aggregation through upregulation of heat shock proteins such as Hsp90, Hsp70, Hsp60, and Hsp40 in mice models [72]. A recent study reported a neuroprotective role of curcumin in an SH-SY5Y cell model of PD against toxic injury by regulating the Hsp90 pathway [73]. Curcumin was also demonstrated to exert a neuroprotective effect in rats who underwent ischemia/reperfusion injury and this effect has been related to the direct scavenger effect of curcumin as well as to a curcumin-induced interference with the apoptotic machinery [74]. In addition, curcumin increased antioxidant molecules GSH and enzymes such as CAT and SOD [75]. It was found that curcumin alleviated the manifestations of NDs by scavenging ROS, disrupting amyloid plaques, and exhibiting anti-inflammatory and anti-apoptotic effects [76]. The impact of curcumin on neural stems cells and other neural cells is quite interesting. Several experimental studies have been published on the capacity of curcumin to affect neural stem cells. While these studies have used a range of neural stem cells, i.e., day 15 embryonic cortex from Sprague Dawley rats [77], rodent spinal cord stem cells [78], SVZ brain region from young Wistar rats [79], C17.2 cells from the cerebellum of neonatal mice, four days old [80]. All of them displayed hormetic-like biphasic dose responses. While there was some variation with respect to the optimal dose, the responses of the four models were remarkably similar. In addition, there was some degree of inter-study variation for the maximum stimulation, with this ranging from approximately 115% to 190%. The width of the stimulation was also somewhat variable, ranging from a low being less than 5-fold to about 25-fold. Limited mechanistic follow-up investigations revealed that curcumin activated the p3 MAP kinase and MEK/pathways [78,80]. It is important to note that the concentration responses of the neural stem cells were also similar to those of non-neural stem cells such as reported for human mesenchymal stem cells [81] and mouse 3T3-L1 preadipocytes [82]. The above findings with neural stem cells are highly consistent with the results of other neuronal biological model experimental systems. These studies include a wide range of non-stem nerve cell responses, such as the effects of curcumin on copper-induced cell oxidative stress [83], striatal cell viability [84] and cell proliferation in PC12 cells [85] and Schwmann cells [86]. Curcumin also protected neurons from beta amyloid [87,88,89,90,91]. In a complementary fashion, Lim et al. (2001) [92] reported that curcumin was protective against amyloid pathology in an Alzheimer’s transgenic mouse models. Likewise, curcumin was found to prevent rotenone- and salsolinol-induced toxicity in a Parkinson’s disease model [93].

## 5. Curcumin, Hormesis and Neuroinflammation

The dose response is central to biology, toxicology, medicine and public health. The dose response often reflects the underlying biology of cells, revealing processes of activation, toxicity and repair. To be better understood, the dose-response needs to be assessed overtime, since living entities are not static but respond to various types of information, such as chemical and physical signals, as well as damage. The dose response, therefore, is dynamic and should be seen as a dose-time-effect process. This is often seen in the context of adaptive responses. Within such a framework, the dose response often displays a dynamically changing biphasic dose response that reflects the process of hormesis.

Hormesis is a biphasic dose response that displays a low-dose stimulation and a high-dose inhibition. It can occur as a result of direct stimulation or as an overcompensation to a disruption in homeostasis [94,95]. In the case of the overcompensation stimulation type of hormesis, this process initially displays an inhibition/toxicity reflected in a threshold or linear dose response. However, the compensatory response, which is usually most effective at lower doses, eventually displays a biphasic dose response at later time points. Regardless of whether the resulting biphasic dose response occurs via direct stimulation or via the overcompensation stimulation, the quantitative features are the same. That is, the low dose stimulation has been shown to be invariably modest, with most of the maximum stimulatory responses being only about 30–60% greater than the control group. The fact that the maximum stimulation is consistently shown to be modest and independent of the mode of stimulation strongly suggests that the hormetic dose response provides a quantitative description of the limits of biological plasticity. In fact, it has been reported that the quantitatively features of the hormetic dose response are also independent of mechanism [96]. That is, it is independent of the receptor and cell signaling pathway, clearly suggesting a very general strategy for biological resource management in the integration of vast signaling and repair process activities.

The hormetic dose response may be typically observed for highly integrative biological endpoints, such as a growth, fecundity, tissue repair, memory, longevity and numerous endpoints. All tend to conform to the quantitative features of the hermetic dose response. The features may also extend to the field of preconditioning/adaptive responses where the vast majority of observations revealed a hormetic dose response [97,98]. Other endpoints, such as chemical-induced hair growth, are also uniformly hermetic. [99] has revealed that the concept of hormesis is extremely generalizable, being independent of biological model, endpoint point, inducing agent, age or gender, potency of the inducing agent and mechanism. Therefore, the hormetic dose response has been highly conserved within an evolutionary framework and has vast biological, biomedical and medical implications. These implications extend to affect essentially all the pharmacological agents that are designed to enhance biological performance, such as increasing memory, strengthening bones, reducing damage from heart attacks and strokes and reducing age-related damage to multiple systems. The fact that hormesis is central to biology and human adaptive and repair responses makes it a key factor affecting how pharmaceutical companies study and assess new drugs. For example, essentially all anxiolytic, anti-seizure and memory-enhancing drugs display hormetic dose responses. This means that the most that one can expect to improve functionality is only within the 30–60% range. These agents are constrained to act within the limits of biological plasticity, which is described by the hormetic dose-response. Therefore, the situation will strongly influence how potential therapeutic agents are studied, affecting the study design, that is, the number of doses, the dose range, and the sample size. This situation also applies to the field of phytochemicals and functional foods, which have the potential to significantly affect biological systems and health outcomes.

A recent report of 99 herbal extracts indicated that most displayed hormetic dose responses in the assessment of anti-inflammatory endpoints using two immune cell systems. In fact, all the biphasic dose responses conformed to the well-recognized features of the hormetic dose response [100]. Amongst the numerous herbal extracts assessed in that study was curcumin, which also displayed a hormetic-like biphasic dose response features. Despite the fact that curcumin has been widely studied and used widely in dietary practices, it only has been recently begun to be seen within a general hormetic framework. The diversity of curcumin-induced hormetic dose response reveals it to be highly pleiotropic, affecting a widely diverse range of such responses.

### Heme Oxygenase as a Pharmacodynamic Paradigm for Curcumin-Related Hormetic Responses

Heme oxygenase is a microsomal enzyme that catalyzes the degradation of heme in a multistep, energy-requiring system [101,102]. The reaction catalyzed by HO is the α-specific oxidative cleavage of heme moieties to form equimolar amounts of ferrous iron, carbon monoxide (CO) and biliverdin (BV); this latter is then reduced by the cytosolic enzyme biliverdin reductase (BVR) into bilirubin (BR), which is then conjugated with glucuronic acid and excreted [101,102,103]. Two main isoforms of HO have been described and called HO-1 and HO-2: HO-1, also referred to as heat shock protein-32, is the redox-sensitive inducible isoform of the HO family, whereas HO-2, the constitutive isozyme, is involved in the physiologic turnover of heme and is also considered as an endogenous probe for gasotransmitters [101,102]. Indeed, *HO-1* is up-regulated by several stimuli, including reactive oxygen and reactive nitrogen species (ROS and RNS, respectively), ischemia-reperfusion, heat shock, LPS, hemin and several drugs [102,104]. *HO-1* induction is one of the earlier cellular responses to tissue damage and is responsible for the antioxidant and neuroprotective features of its by-products [101,102].

Increasing evidence has suggested that the *HO-1* gene is redox-regulated and its promoter contains the antioxidant responsive element (ARE), similarly to other antioxidant enzymes [105].

The induction of *HO-1* is regulated principally by two upstream enhancers, E1 and E2, which contain multiple stress (or antioxidant) responsive elements (StRE, also called ARE) that also conform to the sequence of the Maf recognition element (MARE) [106,107]. There is now evidence to suggest that the heterodimers of Nrf2 and small Maf proteins (i.e., MafK, MafF and MafG) are directly involved in the induction of *HO-1* through these MAREs [106,107]. Under physiological conditions, the Kelch-like ECH-associated protein 1 (Keap1) binds Nrf2, which undergoes ubiquitination and degradation through the proteasome; this is a relatively rapid event, the half-life of Nrf2 degradation being about 3 h [108,109,110,111]. Conversely, under pro-oxidant conditions triggering the cell stress response, Nrf2 is released from Keap1 and migrates into the nucleus, where it binds to conserved ARE/MARE sequence(s) [111,112]. However, since the MARE can be bound by various heterodimeric basic leucine zipper (bZip) factors, including Nrf2 and AP-1, this latter being another *HO-1* inducer [113], this implies the need for fine tuning of the *HO-1* gene transcription in order to avoid any unnecessary up-regulation. This problem could be reconciled by the activity of repressors that prevent non-specific activation. Transcription factor BTB and CNC homology 1 (Bach1) is a transcriptional repressor endowed with DNA binding activity; Bach1-heme interaction is mediated by evolutionarily conserved heme regulatory motifs (HRM), including the cysteine-proline dipeptide sequence [107,114]. Therefore, a reasonable model accounting for the regulation of *HO-1* expression by Bach1 and heme is that *HO-1* gene expression is regulated through antagonism between transcription activators and the repressor Bach1 [107,115]. While under normal physiological conditions, expression of *HO-1* is repressed by the Bach1/Maf complex, increased levels of heme displace Bach1 from the enhancers and allow activators, e.g., heterodimer of Maf with Nrf2, to promote the transcription of *HO-1* gene [107,114,115]. Several lines of evidence have demonstrated the main role played by HO-1 as a target for the neuroprotective effects of curcumin (for extensive and updated reviews on this field, see [116,117]), however, the contribution of the HO-1/BVR system to the hormetic nature of curcumin is worth mentioning. Both the heme degradation activity of HO-1 and the generation of CO and BR should be considered as hormetic events because they induce neuroprotection or neurotoxicity, depending on both concentrations and the cellular redox *milieu*. The heme catabolic activity of HO-1 increases the amount of ferrous iron [Fe(II)] released from the cyclic tetrapyrrole and this reaction is not harmful per se, but if released in excess and in the presence of ROS, Fe(II) is responsible for lipid peroxidation and cell death [118]. Regarding CO, this gasotransmitter has been shown to have neuroprotective outcomes by inhibiting pro-oxidant enzymes (e.g., NADPH oxidase) or activating pro-survival pathways [e.g., the protein kinase B (PKB)/Akt and extracellular signal-related kinase (ERK)/p38 mitogen-activated protein kinase (MAPK)] [102,104,119]. Conversely, CO has been also shown to blunt the systemic anti-inflammatory response via the inhibition of the hypothalamic release of adrenocorticotropin hormone-secretagogues and to activate prostaglandin-endoperoxide synthase, which, in turn, produces pro-inflammatory cytokines [120,121,122,123]. Similarly, BR, a strong antioxidant due to its ability to scavenge both ROS and RNS [124,125,126,127,128] and if produced in excess, impairs brain functions and is responsible for severe diseases, such as *kernicterus* [103,129]. By keeping this in mind, it is possible to support the notion that the over-stimulation of the HO-1/BVR system may be harmful and some cells find it useful to repress HO-1 to avoid toxicity [130,131,132].

The strong link between pharmacodynamics and hormetic responses [133,134] lends support to the dual outcomes reported for curcumin in several diseases [135,136,137]. For instance, curcumin (1–4 g/day for 6 months) increased cholesterol plasma levels in Chinese subjects aged ≥50 years; the mechanism underlying this effect is not known, although the possible interaction of the polyphenol with cholesterol absorption cannot be excluded [138]. The potential interaction of curcumin with drug metabolizing enzymes, such as the cytochrome P450 (CYP) isozymes CYP1A2, CYP2A6, CYP2C6, CYP2C9 and CYP2DS, has been described [117] (see below).

Intriguingly, most of the evidence about the curcumin-HO-1 interaction in the brain describes neuroprotective features [117], and this accounts for the potential adjuvant role of this polyphenol in neurodegenerative disorders [134,139,140].

## 6. Unresolved Issues

Although curcumin is a natural product, this does not imply a lack of safety issues. The toxic effects of curcumin by its interaction with the drug metabolizing enzymes has been recently addressed by Mhillaj et al. [108]. Among the drugs whose plasma levels may undergo toxic fluctuations following curcumin supplementation are clopidogrel, docetaxel, midazolam, nifedepine, norfloxacin and talinolol. The interaction between curcumin and tamoxifen is quite interesting. This latter is an antagonist to estrogen receptors (ER) and is used as an adjuvant therapy in women with breast cancer. Tamoxifen is a pro-drug because the pharmacological effect is due to endoxifen, a metabolite formed through the activities of cytochrome P-450 (CYP) isoforms. In this regard, the administration of curcumin with or without piperine has been shown to inhibit CYP2D6 and CYP3A4 activities, thus reducing tamoxifen antineoplastic effects through the inhibition of endoxifen formation [141]. Furthermore, curcumin has been shown to be responsible for severe liver diseases: the Italian National Institute of Health has reported 19 cases of cholestatic epatitis in subjects assuming curcumin alone or plus piperine as nutritional supplements (www.salute.gov).

These warnings, together with the recommendation by the European Food Scientific Agency (EFSA) dealing with the lack of any scientific evidence strong enough to justify curcumin supplementation in people with inflammatory diseases (e.g., osteoarthritis, rheumatoid arthritis, etc.), raise the question of an unnecessary use of curcumin both in healthy subjects and the elderly [142].

## 7. Future Perspectives and Conclusions

These findings demonstrate that curcumin affects a broad range of cell types in a manner that is consistent with the hormetic-biphasic dose response. This is a case of whether the cells are neural stem, non-neural stem or other cell types. This is also the case with respect to the endpoint measured. This means that the capacity of curcumin to enhance biological performance is constrained by the limits of plasticity and does so in a hormetic fashion. While these findings provide strong evidence that curcumin commonly acts hormetically, the evidence also suggests that there is a limited concentration range within which the hormetic response occurs. However, the limited concentration range generally observed were reported, for the most part, within the context of in vitro studies. It is likely that the range of optimal concentration may be considerably broaden within a whole animal context, even more so within a human framework wherein there is considerable inter-individual variation.

With regard to the potential clinical efficacy of curcumin, there are several pre-clinical data in the literature confirming that curcumin possesses neuroprotective and cognitive-enhancing properties that may help delay or prevent neurodegenerative diseases. There is significant evidence indicating that curcumin can act on multiple pathways identified in the pathogenesis of ND, in particular, curcumin influences AD Aβ aggregation and Aβ clearance, enhancing innate immune systems and reducing oxidative stress, improving cognition and delaying the onset of AD [143]. Although clinical literature data does not presently provide full evidence that curcumin is an efficient neuroprotective agent, owing to conflicting data, however, it is possible that the lack of results on its therapeutic effect is due to limited bioavailability: Baum et al. [138] demonstrated a therapeutic effect of curcumin (1–4 g/day per os for 6 months) on memory and other cognitive skills in subjects with AD. Concerning amyotrophic lateral sclerosis (ALS), recent clinical trials have demonstrated that either curcumin (600 mg/day per os for 3–6 months) or nanocurcumin (80 mg/day per os for 12 months) have antioxidant effects in ALS patients vis-à-vis with the lack of any significant effect in terms of improvement of motor function and other functional measures [144,145]. According to recent evidence using nano-formulation of Curcumin [146], promising outcomes were unraveled suggesting that nanocurcumin, as an adjuvant therapy to riluzole, may have improved the probability of survival in a subgroup of ALS patients with bulbar symptoms [144]. This sustains the conceivable possibility that new therapeutic strategies with nanocurcumin can open up new horizons for very interesting expected results that can be translated in the treatment of PD or MS, as shown in animal studies [147,148,149,150]. Brain function is influenced by the endocrine system, especially by thyroid hormones. Since astrocytes metabolize THs to active form, they play a central role in the endocrine control of neural environment. Oxidative stress is implicated in both hypo- and hyper-thyroid conditions [151]. Further clinical studies, mainly conducted by randomized controlled trials, should be performed to determine the role that curcumin-derived novel delivery systems can play in the prevention and treatment of neuroinflammation.
